# Practice-based observations of barriers to dietary adherence among patients hospitalized with congestive heart failure in a safety-net hospital

**DOI:** 10.3389/fnut.2026.1744773

**Published:** 2026-03-20

**Authors:** Asmaa AlShammari, Anim Asif, Farbod Raiszadeh

**Affiliations:** 1 The Institute of Human Nutrition, Columbia University, New York NY, United States; 2 Kuwait’s Foundation for the Advancement of Sciences, Kuwait, Kuwait; 3 Harlem Hospital Center, Health + Hospitals, Vagelos College of Physicians and Surgeons, Columbia University, New York, NY, United States

**Keywords:** congestive heart failure, health disparities, nutritional barriers, patient perspectives, socioeconomic determinants

## Abstract

Congestive heart failure (CHF) disproportionately affects minority and low-income populations; however, practice-based observations on barriers to dietary adherence remain limited, particularly among African American patients in safety-net settings. This short report presents practice-based observations derived from routine bedside nutrition counseling sessions with 26 adults hospitalized for CHF at a safety-net hospital. Counseling documentation was reviewed and descriptively synthesized to identify recurrent challenges to dietary adherence. Patients frequently demonstrated confusion regarding sodium restriction and nutrition label interpretation and received inconsistent guidance from healthcare providers. Beyond knowledge gaps, socioeconomic and structural barriers, including financial hardship, limited access to healthy foods, reduced mobility, and lack of home support, further impeded dietary adherence. These findings highlight the complex interplay between individual, social, and structural factors that shape nutrition self-management in heart failure. Addressing these barriers through culturally tailored, multidisciplinary interventions that integrate nutrition education with social support and resource linkage may enhance adherence, reduce readmissions, and improve equity in CHF outcomes within underserved populations.

## Introduction

Congestive heart failure (CHF) remains a leading cause of morbidity, mortality, and healthcare utilization in the United States, with a growing burden that is disproportionately borne by low-income and racially minoritized populations (1–3). Recent national data demonstrate rising CHF mortality and hospitalization rates over the past decade, with particularly pronounced increases among African American patients and those residing in socially deprived neighborhoods. African American patients, especially those served by safety-net hospitals, experience disproportionately high rates of CHF readmission due to intersecting social, economic, and healthcare barriers, reflecting persistent inequities in both prevention and disease management (4–10).

Patients in these settings often face intersecting challenges such as financial instability, food insecurity, and limited access to outpatient care, all of which hinder long-term disease management (11–13). Recent literature highlights worsening food insecurity and its adverse impact on cardiovascular outcomes, particularly in the post–COVID-19 period. Within this context, dietary adherence and health literacy have emerged as key determinants of heart failure outcomes, with lower adherence and limited literacy associated with increased hospitalizations and poorer prognosis, disproportionately affecting marginalized populations.

Beyond individual-level factors, health services research has demonstrated that community-level characteristics, including socioeconomic conditions, access to care, and structural healthcare resources, explain a substantial proportion. Within this context, adherence to dietary recommendations, particularly sodium restriction, remains one of the most difficult self-care behaviors to sustain. Cultural food norms, economic constraints, and low health literacy may further complicate adherence even among patients motivated to improve their health (14, 15).

This report summarizes insights derived from routine dietary counseling sessions conducted as part of a multidisciplinary CHF program at a safety-net hospital. The aim was to document commonly expressed challenges to dietary and lifestyle adherence among hospitalized CHF patients and to generate hypotheses for improving nutrition education and discharge planning in socioeconomically vulnerable populations.

## Settings and data source

These practice-based observations were drawn from a hospital-based CHF registry and patient counseling initiative at Harlem Hospital Center, a tertiary public teaching hospital in New York City. The program aimed to improve quality and continuity of care and collect registry data for adult patients hospitalized with a new diagnosis of CHF or with CHF exacerbation.

Between March 1 and July 31, 2022, 26 adults (≥18 years) with a new or exacerbated CHF diagnosis participated in bedside counseling sessions by a dietitian and nutrition graduate students through referrals from attending cardiologists and the CHF Coordinator. Patients discharged from the emergency department, transferred from non-participating hospitals, unable to provide informed consent due to cognitive or linguistic limitations, or with a final diagnosis other than CHF were excluded. Counseling referrals were based on clinical workflow and availability, resulting in a convenience sample rather than consecutive enrollment.

Nutrition counseling sessions were conducted at the bedside by a clinical dietitian and supervised nutrition graduate students using a shared counseling framework that emphasized sodium restriction, identification of high-sodium foods, nutrition label reading, and practical lifestyle modification strategies tailored to individual patient needs. The counseling team operated under the direct clinical oversight of two attending cardiologists and one physician assistant-certified (PA-C) specializing in cardiology. Sessions were conversational and adaptive, rather than guided by a structured interview protocol, reflecting real-world clinical practice.

During or immediately following each counseling session, team members documented patient-reported challenges, misconceptions, and contextual barriers in contemporaneous clinical notes. These notes were independently reviewed by two investigators and descriptively synthesized through iterative review and consensus discussion to identify recurrent challenges related to dietary and lifestyle adherence, with input from supervising cardiologists to ensure clinical relevance. No audio recordings, verbatim transcripts, or formal qualitative coding procedures were used, consistent with the exploratory, practice-based nature of the initiative. Although counselors received program orientation and used a shared framework, variability in counseling style and documentation is acknowledged as a limitation.

This initiative was conducted as part of routine clinical care and quality improvement activities. The CHF registry and associated data collection were deemed exempt from Institutional Review Board review under 45 CFR 46.104(d)(4)(iii), as they involved the secondary use of identifiable health information collected for healthcare operations, in compliance with applicable privacy regulations. A waiver of informed consent was granted due to minimal risk and the impracticability of obtaining consent for retrospective analysis of counseling documentation.

## Results

Twenty-six patients hospitalized with CHF were included in this practice-based observation. The cohort was predominantly male (61.5%) and Black (84.6%), with most (73.1%) unemployed or retired. Comorbidities and health risks were prevalent: 50.0% had diabetes, 23.1% had chronic kidney disease, 50.0% had a history of smoking, 30.8% had a documented substance use disorder, and 57.7% reported daily alcohol consumption. Notably, 15.4% had been hospitalized more than three times for CHF in the preceding six months (see Table 1 for full demographics).

**Table 1:** Baseline demographic and clinical characteristics of hospitalized patients with congestive heart failure.

Variable	Overall (*N* = 26)
Age, year, median (range, in years)	63.3 (39–80)
Gender	
Male	16 (61.5%)
Female	10 (38.5)
Race*	
Black	22 (84.6%)
Employment status	
Employed	4 (15.4%)
Unemployed	11 (42.3%)
Retired	8 (30.8%)
Unknown	3 (11.5%)
Living condition	
Have a permanent residence	25 (96.2%)
Homeless	1 (3.8%)
Smoking status	
History of smoking	13 (50%)
Current smoking	8 (30.7%)
Drug use	
History of drug use	8 (30.8%)
Opiates	4 (15.4%)
Cocaine	3 (11.5%)
Methadone	1 (3.85%)
Marijuana	1 (3.85%)
UTD	1 (3.85%)
Alcohol use	
Current alcohol use	3 (11.5%)
None	15 (57.7%)
Daily	1 (3.85%)
Weekly	-
Occasionally	1 (3.85%)
Unknown	4 (15.4%)
BMI	
Normal	9 (34.6%)
Overweight	6 (23.1%)
Obese	11 (42.3%)
Pro BNP**	
≤1,000	7 (26.9%)
1,001–3,000	5 (19.2%)
3,001–9,000	10 (38.5%)
>9,000	3 (11.5)
Number of hospital admissions (ha)****	
Hospital admissions for CHF in the past 6 months***	
≤3 has	20 (76.92%)
>3 has	4 (15.4%)
All-time hospitalizations	
<5 has	13 (50%)
>5 has	13 (50%)
Comorbidities	
Type 2 diabetes	13 (50%)
Hypertension	20 (76.9%)
Chronic kidney disease	6 (23.1%)
COPD/asthma	7 (26.9%)
Anemia	1 (3.8%)
Atrial flutter	2 (7.7%)

*Number does not equal 100% because the remaining patients’ races were not listed on EPIC. **Numbers do not add up to 100% because one patient’s Pro BNP lab value was not collected. ***Numbers do not add up to 100% because one patient’s previous hospitalization data was not listed on EPIC. ****Numbers do not add up to 100% because two patients had no information listed on EPIC. Pro-BNP: N-terminal Pro-B-Type Natriuretic Peptide; BMI: Body-Mass-Index.

Recurring barriers to dietary adherence were documented from counseling encounters and are described below. Two major categories emerged: knowledge gaps and socioeconomic or structural constraints.

### Knowledge gaps

Within the domain of knowledge gaps, patients frequently demonstrated confusion regarding sodium restriction and heart-healthy eating. Many assumed that vegetarian or vegan diets were inherently beneficial without considering sodium content or food processing. Limited ability to interpret nutrition labels-particularly serving sizes and sodium values-was commonly observed, and several patients were unaware of hidden sources of sodium in processed or restaurant foods. As one patient remarked, “You know how I know this has salt? Because it tastes good,” illustrating a reliance on sensory cues over nutritional information. Some patients also showed incomplete understanding of the relationship between diet, congestive heart failure symptoms, and comorbid conditions such as diabetes or chronic kidney disease.

### Socioeconomic barriers

Socioeconomic and structural barriers were frequently cited and often represented the primary obstacle to dietary adherence. Patients described financial hardship and limited access to affordable, healthy foods, with reliance on convenience stores or fast food driven by neighborhood food environments and cost constraints. Stigma associated with food assistance programs discouraged participation even among eligible individuals. Physical limitations—including fatigue, pain, and reduced mobility—made grocery shopping and meal preparation difficult, particularly for patients living alone or lacking caregiver support. Competing health priorities, such as substance use disorders or chronic pain, further hindered sustained dietary change. In addition, gaps in healthcare system resources, including limited access to social work services, rehabilitation, or substance use treatment, were identified as barriers to effective post-discharge self-management. Representative patient comments, paraphrased from contemporaneous counseling notes and included for illustrative purposes, included: “*I do not have anyone at home to help me cook, and I cannot stand that long in the kitchen,*” “*The minute I’m out of here, I’m going back to what I was doing before,*” and “*When you have no money, you get what you can*”.

## Discussion

These practice-based observations underscore the complexity of dietary adherence among patients hospitalized with congestive heart failure in an underserved, safety-net hospital. The study was conducted in a healthcare setting serving predominantly low-income, minority populations, where patients frequently face additional challenges, including mental illness, homelessness, poverty, and limited access to healthy food (16). These challenges reflect broader social determinants of health (SDOH), including neighborhood food environments, caregiver availability, and structural resource limitations, which not only hinder effective disease management but also contribute to the persistent underrepresentation of these populations in clinical research.

Knowledge gaps such as confusion about sodium restriction, conflicting advice, and misconceptions about healthy diets were common and likely undermine effective self-management. These findings are consistent with existing literature linking patient education deficits to poorer CHF outcomes (14, 17), and align with prior qualitative studies describing patient confusion regarding dietary recommendations and the role of health literacy in heart failure self-care. Tailored, culturally sensitive nutrition education that considers patients’ health literacy and dietary preferences is essential to improve adherence (18).

However, socioeconomic constraints often outweighed knowledge. Even when patients understood dietary principles, financial hardship and limited access to fresh, affordable foods made adherence difficult. Physical limitations, lack of home support, and competing health priorities further complicated lifestyle change. These observations parallel qualitative and observational studies demonstrating the impact of food insecurity, caregiver availability, and social vulnerability on heart failure self-management and readmission risk (19–22). Notably, food insecurity has been linked to nutritional deficiencies, frailty, and worse clinical outcomes in heart failure, even among patients attempting to adhere to sodium restriction (23).

The most recent American College of Cardiology/American Heart Association (ACC/AHA) CHF guidelines (24) emphasize the importance of addressing these barriers through multidisciplinary care. Two class I recommendations highlight that patients with heart failure should (1) receive multidisciplinary care to facilitate guideline-directed medical therapy (GDMT), address barriers to self-care, reduce rehospitalization, and improve survival; and (2) receive structured education and support to promote effective self-care. Notably, the guidelines explicitly identify low health literacy, food and housing insecurity, and financial limitations as key barriers to optimal heart failure management, reinforcing the clinical relevance of the challenges observed in this study.

To translate these recommendations into practice, multidisciplinary CHF programs should integrate practical, culturally appropriate interventions. Potential strategies include simplified educational tools, pre-discharge meal planning support, direct linkage to food assistance programs, and closer coordination with social workers or community health workers to ensure continuity of care. These strategies align with emerging implementation-focused models that integrate clinical care with social support services in underserved populations, such as medically tailored meals or produce prescription programs (25–29). This has shown promise in improving dietary quality and reducing food insecurity in high-risk populations, although access to dietitians and nutrition-related resources remains uneven in many safety-net settings (25–29).

These findings are derived from non-standardized, practice-based counseling notes rather than formal qualitative interviews. As such, the absence of structured interview guides, audio recordings, or formal qualitative coding introduces potential observer and documentation bias and limits reproducibility and depth of interpretation. Nonetheless, these observations highlight the urgent need for equity-focused, resource-sensitive approaches that integrate education with social and clinical support to improve dietary self-management in CHF.

## Conclusion

Dietary adherence in hospitalized CHF patients in a safety-net setting is shaped by overlapping knowledge, socioeconomic, and structural barriers. Patients identified financial hardship, limited food access, physical limitations, and insufficient social or healthcare system support as key obstacles, often outweighing knowledge deficits. These findings underscore the importance of multidisciplinary, culturally responsive care models that combine nutrition counseling with social support and resource linkage. Future implementation-focused research should evaluate scalable interventions addressing social determinants of health to improve equity and outcomes in heart failure.

## Data Availability

The data analyzed in this study cannot be shared due to IRB stipulations and concerns regarding the privacy and confidentiality of the study participants. Requests to access these datasets should be directed to the corresponding author, FR: raiszadf@nychhc.org.

## References

[1] Malik A. Brito D. Vaqar S. Chhabra L. (2022). Congestive heart failure StatPearls Publishing Treasure Island Available online at: http://www.ncbi.nlm.nih.gov/books/NBK430873

[2] Martin SS Aday AW Allen NB Almarzooq ZI Anderson CAM Arora P . 2025 heart disease and stroke statistics: a report of US and global data from the American Heart Association. Circulation. (2025) 151:e41–e660. doi: 10.1161/CIR.0000000000001303, 39866113 PMC12256702

[3] Reddy YN Borlaug BA. Readmissions in heart failure: it’s more than just the medicine. Mayo Clinic Proc. (2019) 94:1919–21. doi: 10.1016/j.mayocp.2019.08.015 31585573

[4] Berenson RA Paulus RA Kalman NS. Medicare's readmissions-reduction program—a positive alternative. N Engl J Med. (2012) 366:1364–6. doi: 10.1056/NEJMp1201268 22455754

[5] Anderson C Deepak BV Amoateng-Adjepong Y Zarich S. Benefits of comprehensive inpatient education and discharge planning combined with outpatient support in elderly patients with congestive heart failure. Congest Heart Fail. (2005) 11:315–21. doi: 10.1111/j.1527-5299.2005.04458.x, 16330907

[6] Ziaeian B Fonarow GC. The prevention of hospital readmissions in heart failure. Prog Cardiovasc Dis. (2016) 58:379–85. doi: 10.1016/j.pcad.2015.09.004, 26432556 PMC4783289

[7] Van Walraven C Bennett C Jennings A Austin PC Forster AJ. Proportion of hospital readmissions deemed avoidable: a systematic review. CMAJ. (2011) 183:E391–402. doi: 10.1503/cmaj.101860 21444623 PMC3080556

[8] Ashton CM Kuykendall DH Johnson ML Wray NP Wu L. The association between the quality of inpatient care and early readmission. Ann Intern Med. (1995) 122:415–21. doi: 10.7326/0003-4819-122-6-199503150-00003, 7856989

[9] Zeng W Chia SY Chan YH Tan SC Low EJ Fong MK. Factors impacting heart failure patients’ knowledge of heart disease and self-care management. Proc Singapore Healthc. (2017) 26:26–34. doi: 10.1177/2010105816664537

[10] Ghali JK. Precipitating factors leading to decompensation of heart failure. Arch Intern Med. (1988) 148:2013. 3046541

[11] Jaarsma T. Nurse led, multidisciplinary intervention in chronic heart failure. Heart. (1999) 81:676. doi: 10.1136/hrt.81.6.676, 10979717 PMC1729056

[12] Annema C Luttik ML Jaarsma T. Reasons for readmission in heart failure: perspectives of patients, caregivers, cardiologists, and heart failure nurses. Heart Lung. (2009) 38:427–34. doi: 10.1016/j.hrtlng.2008.12.002, 19755193

[13] Khan SS Breathett K Braun LT Chow SL Gupta DK Lekavich C . Risk-based primary prevention of heart failure: a scientific statement from the American Heart Association. Circulation. (2025) 151:e1006–26. doi: 10.1161/CIR.0000000000001307, 40235437

[14] Aggarwal M Grady A Desai D Hartog K Correa L Ostfeld RJ . Successful implementation of healthful nutrition initiatives into hospitals. Am J Med. (2020) 133:19–25. doi: 10.1016/j.amjmed.2019.08.019, 31494109

[15] Juárez-Ramírez C Théodore FL Villalobos A Allen-Leigh B Jiménez-Corona A Nigenda G . The importance of the cultural dimension of food in understanding the lack of adherence to diet regimens among Mayan people with diabetes. Public Health Nutr. (2019) 22:3238–49. doi: 10.1017/S1368980019001940, 31385561 PMC10260649

[16] Wojda A Janczy A Małgorzewicz S. Mediterranean, vegetarian and vegan diets as practical outtakes of EAS and ACC/AHA recommendations for lowering lipid profile. Acta Biochim Pol. (2021) 68:41–8. doi: 10.18388/abp.2020_5515, 33544561

[17] Rogerson D Maçãs D Milner M Liu Y Klonizakis M. Contrasting effects of short-term Mediterranean and vegan diets on microvascular function and cholesterol in younger adults: a comparative pilot study. Nutrients. (2018) 10:1897. doi: 10.3390/nu10121897, 30513972 PMC6316028

[18] Nelogal SS Yedam ST Koppula SR Imtiaz H Shettywarangale P Shah B . Chronic disease self-management in heart failure: a narrative review of performance gaps and emerging solutions. Medicine. (2025) 104:e46819. doi: 10.1097/MD.0000000000046819, 41466001 PMC12746952

[19] Scotto CJ. The lived experience of adherence for patients with heart failure. J Cardiopulm Rehabil Prev. (2005) 25:158–63. doi: 10.1097/00008483-200505000-00006, 15931019

[20] Riegel B Carlson B. Facilitators and barriers to heart failure self-care. Patient Educ Couns. (2002) 46:287–95. doi: 10.1016/s0738-3991(01)00165-3, 11932128

[21] Bentley B De Jong MJ Moser DK Peden AR. Factors related to nonadherence to low sodium diet recommendations in heart failure patients. Eur J Cardiovasc Nurs. (2005) 4:331–6. doi: 10.1161/CIR.0000000000001073 15935733

[22] Hahn-Goldberg S Jeffs L Troup A Kubba R Okrainec K. “We are doing it together”; the integral role of caregivers in a patients’ transition home from the medicine unit. PLoS One. (2018) 13:e0197831. doi: 10.1371/journal.pone.0197831, 29795623 PMC5993108

[23] White-Williams C Rossi LP Bittner VA Driscoll A Durant RW Granger BB . Addressing social determinants of health in the care of patients with heart failure: a scientific statement from the American Heart Association. Circulation. (2020) 141:e841–63. 32349541 10.1161/CIR.0000000000000767

[24] Heidenreich P Bozkurt B Aguilar D Allen LA Byun JJ Colvin MM . 2022 AHA/ACC/HFSA guideline for the management of heart failure: a report of the American College of Cardiology/American Heart Association joint committee on clinical practice guidelines. JACC. (2022) 79:e263–421. doi: 10.1016/j.jacc.2021.12.012 35379503

[25] Graven LJ Grant JS. Social support and self-care behaviors in individuals with heart failure: an integrative review. Int J Nurs Stud. (2014) 51:320–33. doi: 10.1016/j.ijnurstu.2013.06.013, 23850389

[26] Schultz WM Kelli HM Lisko JC Varghese T Shen J Sandesara P . Socioeconomic status and cardiovascular outcomes: challenges and interventions. Circulation. (2018) 137:2166–78. doi: 10.1161/CIRCULATIONAHA.117.029652, 29760227 PMC5958918

[27] Mackenbach JP Cavelaars AE Kunst AE Groenhof F. Socioeconomic inequalities in cardiovascular disease mortality. An international study. Eur Heart J. (2000) 21:1141–51. doi: 10.1053/euhj.1999.1990, 10924297

[28] Dimala CA Reggio C Khalife W Donato A. Heart disease and heart failure: trends and disparities in mortality rates in the United States from 2000 to 2020. Am Heart J Plus. (2024) 46:100459. doi: 10.1016/j.ahjo.2024.100459, 39310053 PMC11415632

[29] Bibbins-Domingo K Pletcher MJ Lin F Vittinghoff E Gardin JM Arynchyn A . Racial differences in incident heart failure among young adults. N Engl J Med. (2009) 360:1179–90. doi: 10.1056/NEJMoa0807265, 19297571 PMC2829671

